# The discriminative ability of the triglyceride-glucose index to identify metabolic syndrome among adults of the northern Sri Lankan population

**DOI:** 10.1186/s12902-024-01632-2

**Published:** 2024-07-01

**Authors:** Thurka Paramanathan, Balakumar Sandrasegarampillai, Vasanthy Arasaratnam, Kumanan Thirunavukarasu

**Affiliations:** 1 Department of Biochemistry, Faculty of Medicine, University of Jaffna, Jaffna, Sri Lanka; 2 Department of Medicine, Faculty of Medicine, University of Jaffna, Jaffna, Sri Lanka

**Keywords:** Triglyceride-glucose (TyG) index, Metabolic syndrome, Prevalence, Sri Lanka

## Abstract

**Background:**

The triglyceride-glucose index (TyG index) is a simple surrogate marker for Insulin Resistance (IR). However, the relationship between the TyG index and Metabolic Syndrome (MetS) remains unknown in the Northern Sri Lankan population.

**Methods:**

This was a descriptive, cross-sectional study of adults aged between 18 and 65 years living in Jaffna, Sri Lanka. This study aimed to verify the discriminative ability of the TyG index to identify MetS using the International Diabetes Federation (IDF-2006) criteria and to determine the gender-specific TyG index cut-off values for better prediction of MetS in Northern Sri Lankan adults. TyG index was calculated as Ln[Triglycerides (TG) (mg/dl) × Fasting plasma glucose (FPG) (mg/dl)/2].

**Results:**

A total of 540 individuals were included in this study, with a mean age of 42.18 (± 13.89) years for males and 43.80 (± 12.56) years for females. The mean value of the TyG index in the total study population was 8.54 (± 0.53). Individuals in the higher quartiles of the TyG index had a significantly increased risk of MetS compared with those in the lowest quartile (*p* < 0.01). TyG index showed a stronger association with MetS than the FPG and all the conventional lipid components and the unadjusted odds ratio was 5.47. The area under the curve (AUC) of ROC revealed values of 0.914 (95% confidence interval (CI): 0.884, 0.944) for females, 0.881 (95% CI: 0.830, 0.932) for males and 0.897 (95% CI: 0.870, 0.924) for the total study population. TyG index had a stronger discriminative ability to identify MetS as per IDF criteria in the study population with a cut-off value of 8.60. The mean level of the TyG index significantly increased with the increasing number of MetS components.

**Conclusions:**

The mean value of the TyG index increased as the number of MetS components in the study population increased. Individuals with a higher TyG index had a significantly increased risk of having MetS compared with the lowest quartile of the TyG index. TyG index had a good discriminative ability to diagnose MetS as per IDF criteria among the northern Sri Lankan population.

**Supplementary Information:**

The online version contains supplementary material available at 10.1186/s12902-024-01632-2.

## Background

MetS, characterized by abdominal obesity, hyperglycemia, hypertension, hypertriglyceridemia, and low High-density lipoprotein cholesterol (HDL-C) has become a global health issue that exponentially increases the risk of cardiovascular disease (CVD) [1]. Depending on the number of components of MetS present, individuals with MetS have a higher probability of developing type 2 diabetes mellitus (T2DM) and/or CVD [1]. MetS is anticipated to increase the risk of developing T2DM and CVD by five and two times, respectively, during the next five to ten years [2].

The prevalence of MetS varies depending on the definition of MetS, region, and the demographics of the study group, such as age, sex, race, and ethnicity [3]. According to estimates from the IDF, globally about one-quarter of people have MetS with an estimated prevalence of 20–25% [4]. The prevalence of MetS in Asia Pacific region was reported as 11.9–37.1%, 26.1% in South Asia, and 27.1% in Sri Lanka [5, 6]. It is concerning that the prevalence of MetS is high and on the rise in both developed and developing countries [5]. Identifying individuals with MetS would help to prevent the increasing morbidity and mortality associated with T2DM and CVD.

IR plays an important role in the pathophysiology of MetS. Even though hyperinsulinemic-euglycemic clamp is the gold standard test for the measurement of IR, it is technically difficult to perform in clinical settings due to economical, ethical, and practical constraints. Various indirect measures of MetS have been suggested. One such marker, the fasting TyG index, a product of TG and FPG has been recently proposed as a simple, low-cost, diagnostic marker to identify MetS [3, 7–11]. The TyG index has superiority in identifying IR since it incorporates TG and FPG. However, limited data are available regarding the TyG index as a marker of IR, CVD, and MetS, and almost no literature from Sri Lanka.

Although the prevalence of MetS is significantly rising, awareness and attention given to early detection of MetS are inadequate in the Sri Lankan context. Even though a few population-based studies have proposed the TyG index as an indicator of MetS, no study has examined the discriminative ability of the TyG index for MetS in the Sri Lankan population to date. Since the definition of MetS is ethnic-specific, various populations adopt different MetS criteria and differ in the range of lipid parameters [1], it is necessary to validate the TyG index in each population and determine the cut-off values to be used for the detection of MetS. The TyG index, calculated from low-cost measurements of TG and FPG, is applicable for diagnosing and screening MetS in clinical and population-based studies. Therefore, this study was intended to evaluate the performance of the TyG index to identify MetS using IDF criteria and to determine the TyG index cut-off point for the diagnosis of MetS among adults of the northern Sri Lankan population.

## Methods

### Study population

This cross-sectional study was carried out between June 2013 to August 2013 with a total of 540 community-dwelling adults (165 males and 375 females) aged between 18 and 65 years. Participants were selected by random cluster sampling from four distinct areas of the Jaffna peninsula. All subjects voluntarily participated in the study. Written informed consent was obtained from all the participants of the study and the ethical clearance was obtained from Regional Directorate of Health Services, Jaffna, Sri Lanka.

### Data collection

#### Sociodemographic assessment

A health examination was performed, and a proper medical history was taken by a medical professional. Relevant data such as demographic factors, socio-economic factors, family history, and other ascertained risk factors and medical information was collected using an interviewer-administrated structured questionnaire which was prepared by the investigators of this study.

#### Anthropometric assessment and blood pressure measurement

Anthropometric measurements and blood pressure were recorded using standard protocols in all the participants by trained medical personnel. Body weight was measured with light clothing without shoes by using a calibrated balance beam scale, and height was measured while standing upright and looking straight ahead by using a calibrated stadiometer. Waist circumference (WC) was taken by positioning the non-elastic measuring tape midway between the lower rib margin and the iliac crest, at the end of a normal expiration [3].

Blood pressure was taken while the subject was sitting and rested for at least 5 min. The measurement was taken using the supported right arm at the heart level, using a mercury sphygmomanometer. The mean value of two readings taken ten minutes apart was recorded. In the event of variation of over 20 mmHg between readings, a third reading was done and the mean of the last two readings was used.

#### Biochemical assessment

Blood samples were collected by experienced nursing staff after an overnight fasting in the morning. Peripheral venous blood samples were collected in EDTA-containing tubes and plain serum tubes and stored in an ice box at a temperature of 2–8 °C and the samples were delivered to the laboratory within 2 h after blood collection. FPG, HDL-C, Total cholesterol (TC), and TG were analyzed by the enzymatic colorimetric assay (Erba fully automated analyzer). Low-density lipoprotein cholesterol (LDL − C) level was determined using the Freidwald formula as follows: LDL − C (mg/dl) = TC (mg/dl)—(HDL − C (mg/dl) + (TG (mg/dl)/ 5)) [12]. TyG index, a product of TG and FPG was calculated as Ln[TG (mg/dl) × FPG (mg/dl)/2] for the study population [7]. Body mass index (BMI) was calculated as weight in kilograms divided by height in meters squared (kg/m^2^).

#### Definition of MetS

The IDF criteria of MetS requires central obesity (WC ≥ 80 cm for South Asian women and ≥ 90 cm for South Asian men) as mandatory and at least two of the following components: (1) TG ≥ 150 mg/dl or treatment for hypertriglyceridemia, (2)HDL-C ≤ 50 mg/dl for women and ≤ 40 mg/dl for men, or receiving drug treatment for low HDL-C (3)FPG ≥ 100 mg/dl or previously diagnosed diabetes, (4)Systolic blood pressure (SBP)/ Diastolic blood pressure (DBP) ≥ 130/85 mmHg or receiving drug treatment for previously diagnosed hypertension [4].

#### Statistical analysis

All data were double-entered and cross-checked for consistency. The data was cleaned by checking outliers and missing values. Statistical analyses were performed using SPSS statistical software (version 26.0). All statistical tests were two-tailed and were considered significant for *p* < 0.05. A descriptive analysis of the basic characteristics was performed. Normality was checked for continuous variables using Kolmogorov- Smirnov test. Continuous variables were described as mean and standard deviation (SD). Baseline variables were compared using the independent sample T-test, while categorical variables were compared using the chi-square test among both genders, IDF-defined MetS, and non-MetS groups.

Pearson correlation analysis was used to correlate the TyG index with BMI and WC. Univariate logistic regression analyses were used to evaluate the association between the TyG index and all its components. All the participants were categorized into four age groups (18–29, 30–41, 42–53, and 54–65). The total participants were further classified into four groups according to the TyG index quartiles (≤ 8.1501, 8.1502 to 8.5119, 8.5120 to 8.9307, ≥ 8.9308) and all the MetS components were compared across the quartiles. The odds ratio (OR) along with its 95% CI of MetS were estimated for the higher three quartiles of the TyG index with the lowest one as a reference.

Receiver operating characteristic (ROC) curve analysis was performed with the presence or absence of MetS as per IDF-defined criteria to compare the AUC of TG and FPG with the TyG index and to determine the discriminatory power of the TyG index to identify MetS. An AUC > 0.7 was considered adequate for predicting with acceptable accuracy [3]. Plots of sensitivity (True positive fraction) versus 1 minus specificity (False positive fraction) were constructed for the male, female, and total study population. The cut-off values of the TyG index for MetS as per IDF criteria were established using the Youden index.

## Results

The baseline characteristics of the study population are summarized in Table 1. A total of 540 participants were enrolled in this study of which 175 (32.40%) were males and 365 (67.59%) were females. The mean age (± SD) of the males and females were 42.18 (± 13.89) years and 43.80 (± 12.56) years respectively with the age range of 18 to 65 years. Age, SBP, DBP, and TG were not significantly different among males and females. However, a significant difference in BMI, WC, FPG, TC, HDL-C, LDL-C, and TyG index was observed. In comparison with males, females had significantly higher BMI, WC, TC, HDL-C, and LDL-C (*p* < 0.05) while had lower FPG and the TyG index (*p* < 0.05).

**Table 1:** Baseline characteristics of the study population

	Male	Female	*P*-value
Participant Number (%)	175 (32.40%)	365 (67.59%)	
Age (Years)	42.18 ± 13.89	43.80 ± 12.56	0.193
BMI (kg/m^2^)	22.08 ± 4.09	24.12 ± 5.32	0.000^**^
WC (cm)	91.24 ± 9.11	93.31 ± 10.24	0.023^*^
SBP (mmHg)	122.68 ± 13.12	124.10 ± 14.72	0.277
DBP (mmHg)	78.39 ± 8.83	79.51 ± 8.66	0.163
FPG (mmol/L)	5.35 ± 1.30	5.10 ± 1.26	0.032^*^
TG (mmol/L)	1.44 ± 0.61	1.38 ± 0.65	0.388
TC (mmol/L)	5.09 ± 1.00	5.30 ± 0.93	0.016^*^
HDL-C (mmol/L)	1.20 ± 0.26	1.30 ± 0.26	0.000^**^
LDL-C (mmol/L)	2.99 ± 0.77	3.17 ± 0.74	0.010^*^
TyG index	8.61 ± 0.48	8.50 ± 0.55	0.025^*^
MetS (%)	10.56%	27.22%	0.000^**^

^*^Statistically significant difference between male and female participants

Overall unadjusted prevalence of MetS as per defined IDF criteria in the total study population, males and females were 37.78%, 10.56%, and 27.22% respectively. Females had a significantly higher prevalence than males (*p* < 0.001) **(**Table 1**)**. The mean value of the TyG index in the total study population was 8.54 (± 0.53). TyG index followed a normal distribution in our study population that is advantageous for straightforward interpretation and statistical analysis.

Differences between various anthropometric, and biochemical measures and blood pressure among participants with and without MetS as per IDF criteria are given in Table 2. According to the IDF MetS diagnostic criteria, the TyG index was significantly higher among participants with MetS 8.98 (± 0.38) than Non-MetS 8.27 (± 0.41) (*p* = 0.000). In the regression analysis, individuals with a high TyG index had an increased occurrence of MetS diagnosed by the IDF criteria. As shown in Table 2, MetS participants were more likely to have higher BMI, WC, SBP, DBP, FPG, TG, TC, LDL-C, and TyG index (*p* = 0.000), but lower HDL-C (*p* = 0.000). MetS individuals tended to be older than Non-MetS group (*p* = 0.000).

**Table 2:** Baseline characteristics between the metabolic syndrome (MetS) and non-metabolic syndrome (Non-MetS) groups among both genders

	Male			Female		
	Non-MetS	MetS	*P*-value	Non-MetS	MetS	*P*-value
Participant Number (%)	118(67.43%)	57(32.57%)		218(59.73%)	147(40.27%)	
Age (Years)	40.53 ± 14.19	45.61 ± 12.71	0.000^**^	40.73 ± 12.83	48.35 ± 10.67	0.000^**^
BMI (kg/m^2^)	20.51 ± 3.42	25.33 ± 3.43	0.000^**^	22.56 ± 5.08	26.43 ± 4.81	0.000^**^
WC (cm)	87.67 ± 7.86	98.62 ± 6.81	0.000^**^	90.71 ± 10.20	97.14 ± 9.06	0.000^**^
SBP (mmHg)	118.60 ± 11.72	131.12 ± 11.82	0.000^**^	118.96 ± 12.82	131.73 ± 14.07	0.000^**^
DBP (mmHg)	75.70 ± 8.02	83.95 ± 7.83	0.000^**^	76.53 ± 7.52	83.93 ± 8.34	0.000^**^
FPG (mmol/L)	5.23 ± 1.11	5.61 ± 1.60	0.000^**^	4.69 ± 0.85	5.72 ± 1.49	0.000^**^
TG (mmol/L)	1.17 ± 0.46	1.98 ± 0.53	0.000^**^	1.05 ± 0.39	1.89 ± 0.63	0.000^**^
TC (mmol/L)	4.98 ± 0.9 6	5.40 ± 1.02	0.000^**^	5.16 ± 0.91	5.52 ± 0.93	0.000^**^
HDL-C (mmol/L)	1.27 ± 0.25	1.05 ± 0.22	0.000^**^	1.36 ± 0.24	1.20 ± 0.26	0.000^**^
LDL-C (mmol/L)	2.84 ± 0.73	3.31 ± 0.75	0.000^**^	3.03 ± 0.73	3.38 ± 0.71	0.000^**^
TyG index	8.41 ± 0.41	9.02 ± 0.32	0.000^**^	8.19 ± 0.39	8.97 ± 0.41	0.000^**^

^*^Statistically significant difference between participants with metabolic syndrome and participants without metabolic syndrome

**Fig. 1:**
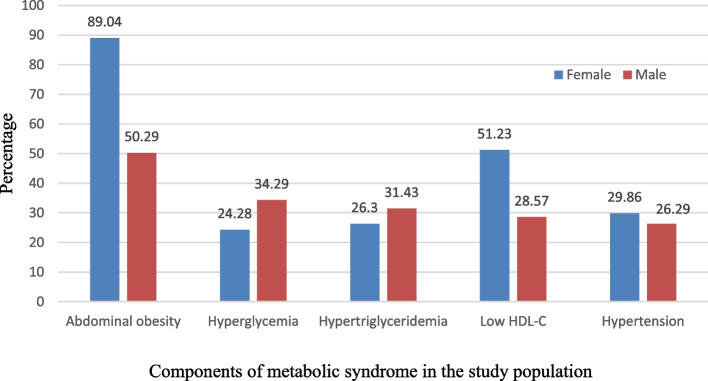
Components of metabolic syndrome in the study population

**Fig. 2:**
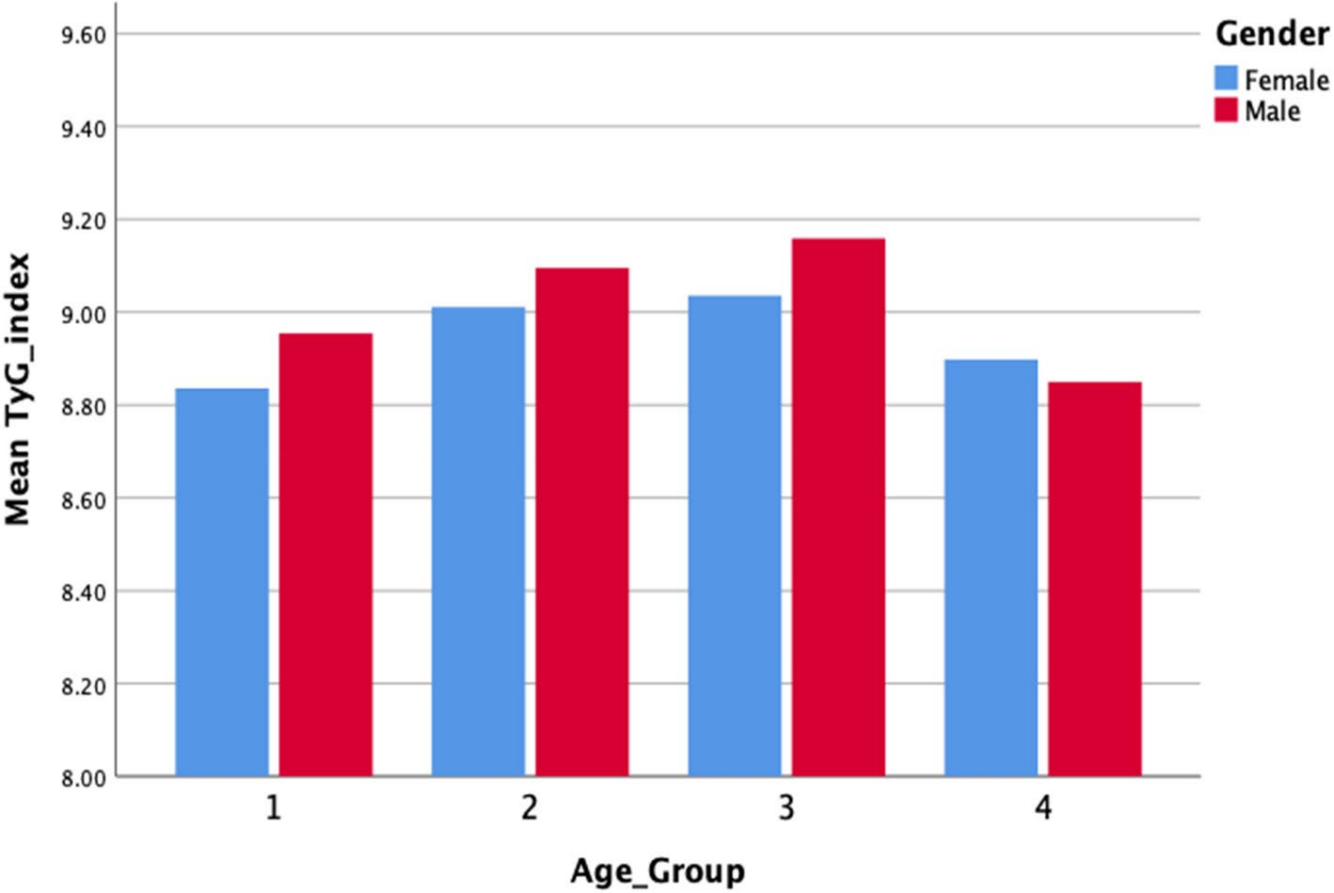
Mean triglyceride- glucose index values between male and females of different age groups with metabolic syndrome; age group 1: 18–23, age group 2: 30–41, age group 3: 42–53 and age group 4: 54–65

**Fig. 3:**
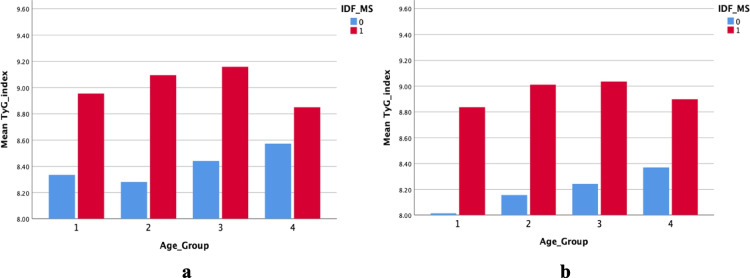
**a** Mean triglyceride- glucose index values of males’ participants with and without metabolic syndrome among different age groups; age group 1: 18–23, age group 2: 30–41, age group 3: 42–53 and age group 4: 54–65. **b** Mean triglyceride- glucose index values of females’ participants with and without metabolic syndrome among different age groups; age group 1: 18–23, age group 2: 30–41, age group 3: 42–53 and age group 4: 54–65

Among the MetS components, abdominal obesity was common (76.48%) among both males and females. Females had a significantly higher risk of having abdominal obesity compared to males (*p* = 0.000). Males were more likely than females to have hyperglycemia. Low levels of HDL-C were more prevalent in females than males. However, there was no significant difference in hypertension, or hypertriglyceridemia between males and females **(**Fig. 1**)**. Age-based grouping of the participants with MetS revealed that the TyG index value did not substantially change with age **(**Fig. 2**)**. TyG index gradually increased among the participants with MetS over the first three age groups, with a little lower value obtained for the age group of 54–65 years. In contrast, those with Non-MetS exhibited a steady increase in the TyG index with ageing **(**Figs. 3a and 3b**)**.

**Fig. 4:**
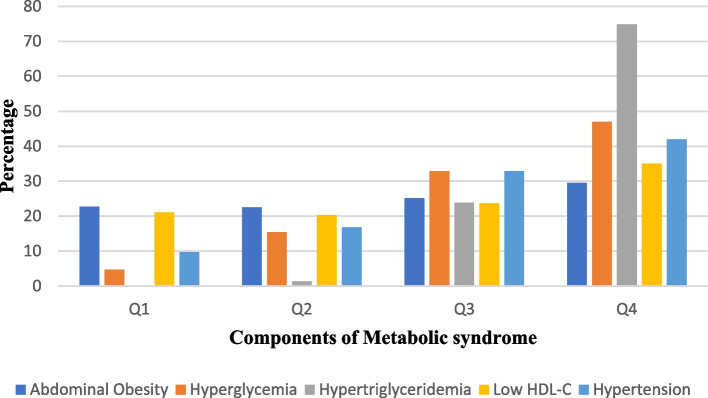
Metabolic syndrome components across triglyceride-glucose index quartiles

**Fig. 5:**
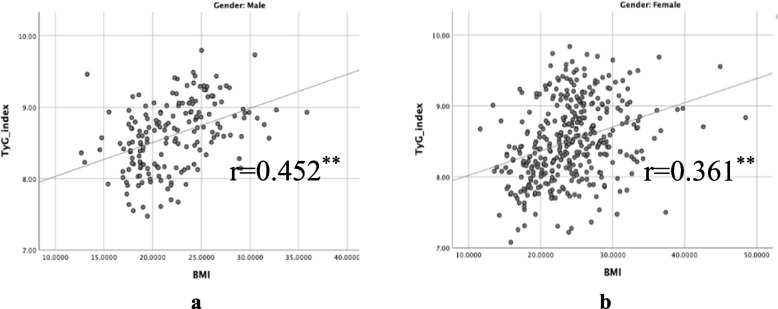
Scatter plot showing significant correlation of triglyceride-glucose index (TyG index) with Body Mass Index (BMI)

**Fig. 6:**
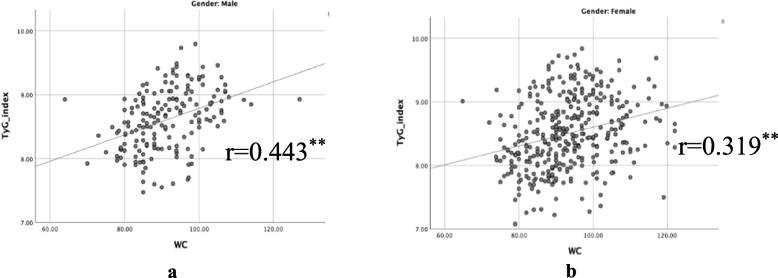
Scatter plot showing significant correlation of Triglyceride-glucose index (TyG index) with Waist Circumference (WC)

As shown in Table 3 and Fig. 4, participants belonging to higher TyG index quartiles had significantly higher BMI, WC, SBP, DBP, FPG, TG, TC, and LDL-C than the participants in the lower TyG index quartiles (*p* = 0.000) but had lower HDL-C (*p* = 0.000). Table 4 gives the percentiles of the TyG index, indicating that the value of the 50th percentile of the TyG index in the MetS group of males agreed with the value of the 95th percentile in the Non-MetS group of males while the 25th percentile of the TyG index in the MetS group of females agreed with the value of 90th percentile in the Non-MetS group of females. In addition, the Pearson correlation analysis of TyG index showed a weak positive linear correlation with anthropometric measures such as BMI and WC **(**Figs. 5 and 6**)**.

**Table 3:** Baseline characteristics in the triglyceride-glucose index quartiles among the study population

	Q1	Q2	Q3	Q4	*P*-value
Participant number	135	135	135	135	
Age (Years)	37.04 ± 12.63	43.33 ± 12.44	45.19 ± 13.84	47.55 ± 10.71	0.000^**^
BMI (kg/m^2^)	21.26 ± 4.36	22.50 ± 4.75	24.64 ± 5.26	25.44 ± 4.69	0.000^**^
WC (cm)	88.45 ± 8.64	91.08 ± 9.70	94.65 ± 10.46	96.39 ± 9.93	0.000^**^
SBP (mmHg)	116.43 ± 11.14	122.00 ± 12.61	125.87 ± 14.90	130.27 ± 14.30	0.000^**^
DBP (mmHg)	75.69 ± 6.97	77.73 ± 8.46	80.56 ± 9.18	82.59 ± 8.57	0.000^**^
FPG (mmol/L)	4.50 ± 0.69	4.79 ± 0.88	5.34 ± 0.96	6.11 ± 1.68	0.000^**^
TG (mmol/L)	0.76 ± 0.18	1.13 ± 0.25	1.50 ± 0.32	2.22 ± 0.52	0.000^**^
TC (mmol/L)	4.76 ± 0.78	5.25 ± 0.92	5.32 ± 0.95	5.59 ± 1.00	0.000^**^
HDL-C (mmol/L)	1.34 ± 0.23	1.34 ± 0.28	1.24 ± 0.25	1.15 ± 0.25	0.000^**^
LDL-C (mmol/L)	2.75 ± 0.65	3.11 ± 0.72	3.21 ± 0.71	3.37 ± 0.78	0.000^**^

*Q1* Quartile 1, *Q2* Quartile 2, *Q3* Quartile 3 and *Q4* Quartile 4

^*^Statistically significant difference across Triglyceride- glucose index quartiles

**Table 4:** Distribution of the triglyceride-glucose index by gender

	Percentiles of the triglyceride-glucose index											
	10th	20th	25th	30th	40th	50th	60th	70th	75th	80th	90th	95th
Non-MetS												
Both	7.75	7.91	7.99	8.06	8.16	8.25	8.36	8.49	8.55	8.61	8.81	8.93
Male	7.91	8.04	8.13	8.15	8.26	8.41	8.51	8.67	8.71	8.78	8.93	9.06
Female	7.72	7.84	7.91	8.00	8.09	8.20	8.29	8.38	8.43	8.50	8.69	8.83
MetS												
Both	8.49	8.67	8.74	8.79	8.92	9.02	9.11	9.20	9.25	9.30	9.45	9.58
Male	8.61	8.76	8.81	8.85	8.93	9.05	9.07	9.20	9.27	9.29	9.44	9.51
Female	8.43	8.62	8.71	8.75	8.91	9.01	9.11	9.20	9.24	9.32	9.48	9.61

The results of binary logistic regression analysis using the dichotomous variable ‘MetS’ (0 = absent, 1 = present) as the dependent variable showed a significant association between other independent variables such as TyG index, lipid profile, FPG, BMI, WC, SBP, and DBP (*p* = 0.000). In addition, the odds ratio (OR) for MetS increased significantly with increasing levels of the TyG index. Individuals with higher TyG index had a significantly increased risk of MetS compared with the reference group with ORs of 3.00, 10.33, and 19.67 respectively (*p* = 0.000) **(**Table 5**)**.

**Table 5:** Odds ratios (95% confidence interval) of metabolic syndrome according to lipid components and risk factors

	Unadjusted odds ratio	95% Confidence interval	*P*-value
BMI	1.226	1.171 – 1.284	0.000^**^
WC	1.097	1.073 – 1.121	0.000^**^
SBP	1.079	1.061 – 1.097	0.000^**^
DBP	1.130	1.100 – 1.160	0.000^**^
FPG	1.796	1.505 – 2.145	0.000^**^
TG	2.735	2.285 – 3.273	0.000^**^
TC	1.574	1.301 – 1.905	0.000^**^
HDL-C	0.054	0.024 – 0.119	0.000^**^
LDL-C	2.084	1.623 – 2.676	0.000^**^
TyG index	5.470	4.102 –7.294	0.000^**^
TyG index quartiles			
≤ 8.1501	1.000 (Reference)		
8.1502 to 8.5119	3.000	1.191 – 7.558	0.014^*^
8.5120 to 8.9307	10.333	4.470 – 23.887	0.000^**^
≥ 8.9308	19.667	8.660 –44.664	0.000^**^

^*^Statistically significant association between the selected parameters and the metabolic syndrome

**Fig. 7:**
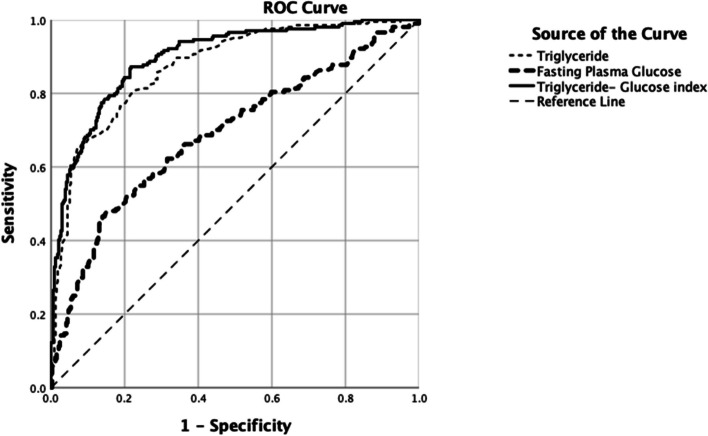
The discriminatory power of the triglyceride-glucose index (TyG index) for metabolic syndrome by receiver operating characteristics (ROC) curve

**Fig. 8:**
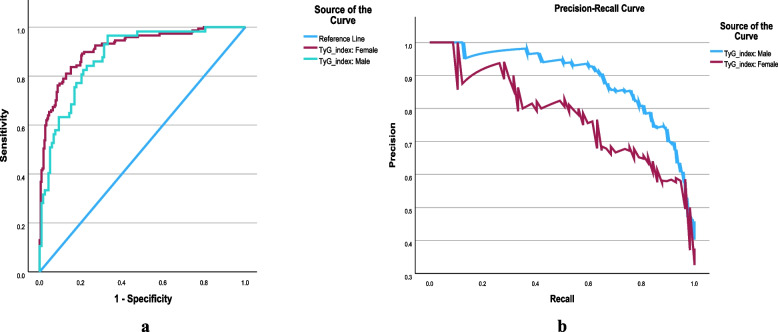
The discriminatory power of the Triglyceride-glucose (TyG index) for Metabolic syndrome by receiver operating characteristics (ROC) curve and precision-recall curve (PRC) in both gender

The analysis of the ROC curve showed that the TyG index had a stronger discriminative ability to identify MetS in females compared with males. The AUC of ROC with TyG index revealed values of 0.914 (CI: 0.884, 0.944) for females, 0.881 (CI: 0.830, 0.932) for males, and 0.897 (CI: 0.870, 0.924) for the total study population, which indicates TyG index has moderate to high accuracy for MetS screening **(**Figs. 7a & 7b**)**. As shown in Fig. 8, the mean value of the TyG index increased as the number of MetS components in the study population increased (*p* = 0.000).

**Fig. 9:**
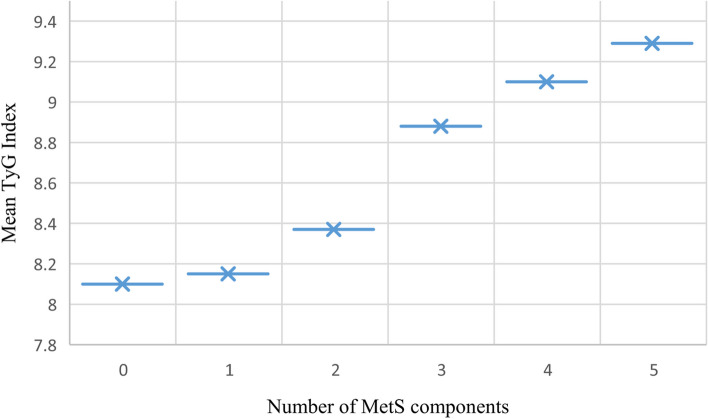
Triglyceride-glucose index (TyG index) as related to the number of components of metabolic syndrome in the overall study population

As shown in Table 6** and **Fig. 9, the TyG index can serve as a more accurate marker for the diagnosis of MetS because it had the largest AUC in comparison to FPG (0.689, 95% CI: 0.642, 0.736) and TG (0.874, 95% CI: 0.844, 0.904). Although ROC curve analysis revealed different TyG index cut-off values for males and females (8.76 vs 8.48), cut-off values were not significantly different than that of the total study population (8.60).

**Table 6:** The area under the receiver operating characteristic curve and the cut-off values of the TG, FPG, and TyG index for identifying metabolic syndrome

Variable	AUC (95% CI)	Cut-off value	Sensitivity %	Specificity %
TG	0.874 (0.844, 0.904)	1.34	80.40	77.40
FPG	0.689 (0.642, 0.736)	5.03	67.20	61.60
TyG index:				
Both	0.897 (0.870, 0.924)	8.60	84.30	81.10
Male	0.881 (0.830, 0.932)	8.76	80.70	79.70
Female	0.914 (0.884, 0.944)	8.48	89.10	79.98

## Discussion

In this observational study, our findings indicated that individuals with a higher TyG index tended to have a higher risk of MetS. The TyG index, calculated as Ln[TG (mg/dl) × FPG (mg/dl)/2], was constructed as a marker of IR and has been proven to be significantly correlated with HOMA-IR [3, 7, 8, 13]. An association between the TyG index and MetS has been shown in a few studies so far [3, 7, 13, 14]. Early diagnosis and timely intervention of MetS are necessary to reduce the incidence of T2DM and CVD. IR, which is the key underlying factor is technically difficult to measure in general practice or during large population-based studies. Therefore, validating a surrogate marker of MetS like the TyG index in our population is extremely beneficial.

The present study used the definition proposed by the IDF to define MetS. IDF definition uses ethnic-specific criteria for WC, including distinct cut-off points for the South Asian population. The application of other MetS definitions such as NCEP-ATP III in Asian populations results in a lower prevalence of MetS, which is incompatible with the actual higher prevalence of T2DM and CVDs [6]. This is mainly due to unspecified WC cut-off values for the non-Europid population proposed by NCEP-ATP III. Evidence has shown that the WHO definition of MetS, in which IR plays a central role, is better predicted than the NCEP-ATP III who are at high risk of getting CVD [15].

Nearly one-fourth of individuals worldwide, according to estimates from the IDF, have MetS [1]. In most nations, the prevalence of MetS is rising in tandem with the twin global epidemics of T2DM and obesity. Previous studies have estimated a MetS prevalence of 24.3% among adults in Sri Lanka [6]. Central obesity, as measured by WC is one of the major determinants of MetS [16]. Our analysis showed that the increase in BMI and WC accounted for much of the increase in the prevalence of the MetS in females than males **(**Table 1 and Fig. 3**)**. The risk of being centrally obese was higher in females than males. Pearson correlation analyses showed a positive correlation between the TyG index with obesity indicative anthropometric indices such as WC and BMI. A higher prevalence of MetS in females may be partly attributed to the use of lower WC cut-off values to determine abdominal obesity to define MetS as per defined IDF criteria.

MetS individuals tended to be older than Non-MetS group. It should come as no surprise that the MetS increases in frequency with each decade of life, paralleling age-related increases in obesity and, particularly, central adiposity. Studies revealed that the prevalence of MetS continued to rise with age into the sixth decade, with women’s prevalence eventually overtaking men’s after they turned 60 years of age [6]. These patterns imply that the prevalence of MetS is influenced by both age and sex. Despite the fact that ageing affects MetS, TyG index was not significantly different among people with MetS across different age groups.

Early diagnosis of MetS is essential to prevent its long-term consequences such as T2DM and CVD. In this study, we determined the discriminative ability of the TyG index to identify MetS and calculated the gender-specific cut-off values of the TyG index (8.48 in females, 8.76 in males, and 8.60 in the total study population) which are in par with the previously published studies [3, 13]. Sex stratification may be viewed as unnecessary because cut-off values of the TyG index for differentiating MetS in males and females observed in this study (8.76 and 8.48) did not substantially differ from those of the total study population (8.60), which is important for its usage in routine clinical practice. In this sense, the TyG index of 8.60 derived without sex stratification can be used as the cut-off value with the maximum sensitivity and specificity to distinguish MetS. Despite different MetS definitions used, previous studies conducted in different parts of the world have reported the approximately same cut-off values for the TyG index to discriminate MetS in their population [7, 17]

In this study, the AUC of the TyG index was 0.897 for the total population, which confirmed that the TyG index had a good discriminative ability to be used as a sensitive marker to identify MetS. In line with our results, previous studies conducted with different MetS definitions showed that the TyG index was a better marker of MetS than FPG, TG, small dense LDL-C, non-HDL-C, and HOMA- IR, and similar AUC values were obtained for ROC curve analysis. The TyG index has significant advantages over other IR markers, such as HOMA-IR, which are more complex and resource-intensive to measure. HOMA-IR requires fasting insulin levels, which are not commonly available in low-resource settings, making it less practical for widespread use. In contrast, the TyG index relies on readily available tests, making it a more accessible and feasible option.

A simple marker like TyG index makes screening efficient and cost-effective, especially in resource-limited primary healthcare settings in Sri Lanka. The TyG index is a valuable tool for primary care physicians, enabling early and accurate identification of MetS. With an AUC of 0.897, it has a strong discriminative ability, supporting its use as a sensitive marker for MetS. A TyG index cut-off of 8.60 can be used universally, providing a consistent and reliable threshold for MetS diagnosis without the need for gender-specific values. Its correlation with BMI and WC allows physicians to monitor and address central obesity, a key determinant of MetS. Its simplicity, cost-effectiveness, and strong predictive ability make it ideal for routine use in diverse clinical settings, improving the management and outcomes of patients at risk for MetS, T2DM, and CVD.

Different definitions of MetS may prioritize certain components over others. The NCEP-ATP III definition is widely used criteria of MetS and incorporates the key features of hyperglycemia/IR, central obesity, dyslipidemia and hypertension. In contrast, the IDF criteria for MetS utilizes population-specific cut-offs for defining obesity, whereas the NCEP-ATP III definition does not rely on such cut-offs. Additionally, the World Health Organization definition requires an absolute requirement for IR, which can manifest as impaired FPG, impaired glucose tolerance, or HOMA-IR. Despite discrepancies between these definitions, a recent systematic review and meta-analysis demonstrated that the TyG index maintains its discriminative ability across different definitions of MetS. The summary receiver-operating characteristics (sROC) curves for various MetS definitions exhibited an AUC of 0.87 (95 CI: 0.84,0.90), indicating that the TyG index is a sensitive marker for diagnosing MetS across different countries and using different definitions [7]. This suggests that the TyG index remains a valuable tool for identifying MetS regardless of the specific criteria employed, highlighting its robustness and versatility in clinical practice.

Recent studies revealed the TyG index as a crucial marker for cardiovascular risk assessment. Its significance extends particularly to patients with T2DM and CVD, including conditions like stable coronary artery disease (CAD) and acute coronary syndrome. Importantly, studies have highlighted its predictive ability of the onset of atherosclerosis, myocardial infarction, and CAD, showcasing its relevance in the diabetic and the general population. Moreover, investigations have demonstrated strong associations between higher TyG index levels and increased risks of heart failure, stroke, and atrial fibrillation, underscoring its multifaceted role in cardiovascular health. Notably, patients with elevated TyG index levels not only face increased risks of these cardiovascular events but also encounter worse prognoses, marked by higher rates of adverse outcomes, including mortality. This underscores the significance of the TyG index in both risk assessment and prognostic evaluation across various cardiovascular conditions. Moreover, its simplicity as a marker of IR enhances its usefulness in risk stratification and the development of tailored management strategies for CVDs [18–23].

## Strengths and limitations of the study

A few of the benefits of our study are worth highlighting. At first, this is the only study conducted in the adult population in Sri Lanka that proves the superior discriminative ability of the TyG index for MetS over all the biochemical components of MetS. When FPG and TG combined to create a composite of the TyG index for MetS, that would eliminate the need for inconsistent evaluation of various lipid components and make the MetS diagnostic process simpler. Additionally, the results showed the highest association between the TyG index and MetS, as well as the improved predictive ability of the TyG index over FPG and traditional lipids, which were steady and dependable due to high statistical power. Strict quality controls were used in the execution of all data collection and laboratory analysis. The relatively large sample size and the comprehensive analysis of the variables were additional strengths of our study.

As regards the limitations of this study, by comparing the proposed definitions to IR, which is best assessed using the hyperinsulinemic-euglycemic clamp technique or HOMA-IR, the current study may have been made better. To confirm TyG index cut-offs to define MetS, more representative data from different ethnic groups of Sri Lankans with a much larger sample size could have been used. Our study was a cross-sectional study where we could not establish a causal relationship. Further longitudinal studies are suggested on the predictive ability of the TyG index for IR and MetS in the Sri Lankan population.

Despite the limitations we described, we hope this study is still clinically relevant because it offers a relatively straightforward mathematical marker for clinical usage that is not only affordable but also practical in primary healthcare settings with limited laboratory resources. In addition, a simple sensitive marker like the TyG index can be utilized to replace the varying MetS definitions and MetS defining criteria that create diagnostic confusion and complicate otherwise simple situations in primary healthcare settings and epidemiological studies. It is commonly recognized that different classifications utilize different criteria, composition (age groups, gender, WC cut-offs, and ethnicity), degree of urbanization, lifestyle, and other socio-cultural factors which causes the prevalence of MetS to vary. Hence, it’s difficult to make a meaningful comparison of prevalence across countries. However, the TyG index can be used to generalize the outcomes of prevalence studies.

## Conclusions

This study evaluated the discriminative ability of the TyG index as a predictor of MetS and revealed a significant positive association between the TyG index and the risk of MetS. TyG index had a good discriminative ability to identify MetS with the AUC of the ROC curve of 0.897 (0.870, 0.924) and the optimal cut-off value of the TyG index for detecting MetS as per defined IDF criteria in Sri Lankan adults was 8.60 for the total study population, with a sensitivity of 84.30% and specificity of 81.10%. Age and sex stratification are deemed unnecessary. These research findings can be utilized to reduce the cost of MetS screening in primary healthcare settings and population-based studies in resource-poor countries to improve early prediction and identification of individuals with MetS.

### Supplementary Information

Supplementary Material 1.

## Data Availability

The data that supports the findings of the study are available upon reasonable request to the corresponding author.

## Abbreviations

AUC: Area under the curve
BMI: Body mass index
CAD: Coronary artery disease
DBP: Diastolic blood pressure
FPG: Fasting plasma glucose
HDL-C: High-density lipoprotein cholesterol
IDF: International diabetes federation
IR: Insulin resistance
LDL-C: Low-density lipoprotein cholesterol
MetS: Metabolic syndrome
NCEP-ATP III: National Cholesterol Education Program Adult Treatment Panel III
Non-MetS: Non-metabolic syndrome
ROC: Receiver operating characteristics
SBP: Systolic blood pressure
TC: Total cholesterol
TG: Triglyceride
TyG index: Triglyceride-glucose index
WC: Waist circumference

## References

[1] International Diabetes Federation. The IDF consensus worldwide definition of the metabolic syndrome. Belgium: International Diabetes Federation; 2006.

[2] Alberti KGMM, Eckel RH, Grundy SM, Zimmet PZ, Cleeman JI, Donato KA (2009). Harmonizing the metabolic syndrome: A joint interim statement of the international diabetes federation task force on epidemiology and prevention; National heart, lung, and blood institute; American heart association; World heart federation; International atherosclerosis society; And international association for the study of obesity. Circulation.

[3] Couto AN, Pohl HH, Bauer ME, Schwanke CHA. Accuracy of the triglyceride-glucose index as a surrogate marker for identifying metabolic syndrome in non-diabetic individuals. Nutrition 2023;109. 10.1016/j.nut.2023.111978. 10.1016/j.nut.2023.111978 36842288

[4] Ranasinghe P, Mathangasinghe Y, Jayawardena R, Hills AP, Misra A. Prevalence and trends of metabolic syndrome among adults in the Asia-pacific region: a systematic review. BMC Public Health 2017;17. 10.1186/s12889-017-4041-1. 10.1186/s12889-017-4041-1 PMC5251315 28109251

[5] Katulanda P, Ranasinghe P, Jayawardana R, Sheriff R, Matthews DR. Metabolic syndrome among Sri Lankan adults: prevalence, patterns and correlates. Diabetol Metab Syndr. 2012;4(1):24. 10.1186/1758-5996-4-24. 10.1186/1758-5996-4-24 PMC3407762 22650800

[6] Nabipoorashrafi SA, Seyedi SA, Rabizadeh S, Ebrahimi M, Ranjbar SA, Reyhan SK (2022). The accuracy of triglyceride-glucose (TyG) index for the screening of metabolic syndrome in adults: A systematic review and meta-analysis. Nutr Metab Cardiovasc Dis.

[7] Khan SH, Sobia F, Niazi NK, Manzoor SM, Fazal N, Ahmad F. Metabolic clustering of risk factors: evaluation of triglyceride-glucose index (TyG index) for evaluation of insulin resistance. Diabetol Metab Syndr 2018;10. 10.1186/s13098-018-0376-8. 10.1186/s13098-018-0376-8 PMC6173832 30323862

[8] Du T, Yuan G, Zhang M, Zhou X, Sun X, Yu X. Clinical usefulness of lipid ratios, visceral adiposity indicators, and the triglycerides and glucose index as risk markers of insulin resistance. Cardiovasc Diabetol 2014;13. 10.1186/s12933-014-0146-3. 10.1186/s12933-014-0146-3 PMC4209231 25326814

[9] Mohd Nor NS, Lee SJ, Bacha F, Tfayli H, Arslanian S (2016). Triglyceride glucose index as a surrogate measure of insulin sensitivity in obese adolescents with normoglycemia, prediabetes, and type 2 diabetes mellitus: comparison with the hyperinsulinemic–euglycemic clamp. Pediatr Diabetes.

[10] Irace C, Carallo C, Scavelli FB, De Franceschi MS, Esposito T, Tripolino C, et al. Markers of insulin resistance and carotid atherosclerosis. A comparison of the homeostasis model assessment and triglyceride glucose index. Int J Clin Pract 2013;67:665–72. 10.1111/ijcp.12124. 10.1111/ijcp.12124 23758445

[11] Dintshi M, Kone N, Khoza S. Comparison of measured LDL cholesterol with calculated LDL-cholesterol using the Friedewald and Martin-Hopkins formulae in diabetic adults at Charlotte Maxeke Johannesburg academic hospital/NHLS laboratory. PLoS One 2022;17. 10.1371/journal.pone.0277981. 10.1371/journal.pone.0277981 PMC9749991 36516155

[12] Jiang M, Li X, Wu H, Su F, Cao L, Ren X, et al. Triglyceride-Glucose Index for the Diagnosis of Metabolic Syndrome: A Cross-Sectional Study of 298,652 Individuals Receiving a Health Check-Up in China. Int J Endocrinol 2022;2022. 10.1155/2022/3583603. 10.1155/2022/3583603 PMC9259285 35814916

[13] Raimi TH, Dele-Ojo BF, Dada SA, Fadare JO, Ajayi DD, Ajayi EA (2021). Triglyceride-Glucose Index and Related Parameters Predicted Metabolic Syndrome in Nigerians. Metab Syndr Relat Disord.

[14] Misra A, Wasir JS, Pandey RM. An evaluation of candidate definitions of the metabolic syndrome in adult Asian Indians. Diabetes Care. 2005;28(2):398–403. 10.2337/diacare.28.2.398. 10.2337/diacare.28.2.398 15677799

[15] Gasevic D, Frohlich J, Mancini GJ, Lear SA. Clinical usefulness of lipid ratios to identify men and women with metabolic syndrome: A cross-sectional study. Lipids Health Dis 2014;13. 10.1186/1476-511X-13-159. 10.1186/1476-511X-13-159 PMC4210572 25300321

[16] Unger G, Benozzi SF, Perruzza F, Pennacchiotti GL. Triglycerides and glucose index: a useful indicator of insulin resistance. Endocrinol Nutr. 2014;61(10):533–40. 10.1016/j.endonu.2014.06.009. 10.1016/j.endonu.2014.06.009 25174769

[17] Liu X, Tan Z, Huang Y, Zhao H, Liu M, Yu P, et al. Relationship between the triglyceride-glucose index and risk of cardiovascular diseases and mortality in the general population: a systematic review and meta-analysis. Cardiovasc Diabetol 2022;21. 10.1186/s12933-022-01546-0. 10.1186/s12933-022-01546-0 PMC9250255 35778731

[18] Liu F, Ling Q, Xie S, Xu Y, Liu M, Hu Q, et al. Association between triglyceride glucose index and arterial stiffness and coronary artery calcification: a systematic review and exposure-effect meta-analysis. Cardiovasc Diabetol 2023;22. 10.1186/s12933-023-01819-2. 10.1186/s12933-023-01819-2 PMC10183133 37179288

[19] Azarboo A, Behnoush AH, Vaziri Z, Daneshvar MS, Taghvaei A, Jalali A, et al. Assessing the association between triglyceride-glucose index and atrial fibrillation: a systematic review and meta-analysis. Eur J Med Res 2024;29. 10.1186/s40001-024-01716-8. 10.1186/s40001-024-01716-8 PMC10860290 38347644

[20] Khalaji A, Behnoush AH, Khanmohammadi S, Ghanbari Mardasi K, Sharifkashani S, Sahebkar A, et al. triglyceride-glucose index and heart failure: a systematic review and meta-analysis. Cardiovasc Diabetol 2023;22. 10.1186/s12933-023-01973-7. 10.1186/s12933-023-01973-7 PMC10486123 37679763

[21] Liang S, Wang C, Zhang J, Liu Z, Bai Y, Chen Z, et al. Triglyceride-glucose index and coronary artery disease: a systematic review and meta-analysis of risk, severity, and prognosis. Cardiovasc Diabetol 2023;22. 10.1186/s12933-023-01906-4. 10.1186/s12933-023-01906-4 PMC10327356 37415168

[22] Yang Y, Huang X, Wang Y, Leng L, Xu J, Feng L, et al. The impact of triglyceride-glucose index on ischemic stroke: a systematic review and meta-analysis. Cardiovasc Diabetol 2023;22. 10.1186/s12933-022-01732-0. 10.1186/s12933-022-01732-0 PMC9825038 36609319

[23] De Silva ST, Niriella MA, Ediriweera DS, Kottahachchi D, Kasturiratne A, De Silva AP, et al. Incidence and risk factors for metabolic syndrome among urban, adult Sri Lankans: A prospective, 7-year community cohort, follow-up study. Diabetol Metab Syndr 2019;11. 10.1186/s13098-019-0461-7. 10.1186/s13098-019-0461-7 PMC6694684 31428204

