# Supplementary material for: The discriminative ability of the triglyceride-glucose index to identify metabolic syndrome among adults of the northern Sri Lankan population
Source: BMC Endocr Disord. 2024 Jul 1;24:101. doi: 10.1186/s12902-024-01632-2 (PMC11218160; doi:10.1186/s12902-024-01632-2)
Supplement: Supplementary file 1 — Supplementary Material 1. [file 12902_2024_1632_MOESM1_ESM.pdf]

SECTION A: Socio-demographic factors

1. Gender:…………………………………………………….

2. Age at last birthday:………………………………………….

3. Area of residence
   a. Urban ☐
   b. Rural ☐
   c. Not known ☐

SECTION B: Smoking/ Alcohol consumption

1. Smoking
   a. Current smoker ☐
   b. Former smoker ☐
   c. Never smoker ☐

2. Alcohol consumption
   a. Current drinker ☐
   b. Former drinker ☐
   c. Never drinker ☐

SECTION C: Medical conditions

**Medical conditions**	**Present**	**Absent**
Diabetes mellitus		
Dyslipidemia		
Hypertension		
Ischemic heart disease		
Stroke		
Coronary Heart Disease		
Hyperthyroidism		
Hypothyroidism		

SECTION D: Medications

**Drugs**	**Yes**	**No**
Statin		
Hypertensive Drugs		
Insulin		
Metformin		
Asprin		
Levothyroxine		

SECTION E: Family history of the selected medical condition

Family history of Obesity

a. Yes ☐<br>i. 1st degree relations (Parents, Siblings, Child) ☐<br>ii. 2nd degree Relations (Grandparents, Aunt, Uncle, Grandchild) ☐	b. No ☐

Family history of Hypertension

a. Yes ☐<br>i. 1st degree relations (Parents, Siblings, Child) ☐<br>ii. 2nd degree Relations (Grandparents, Aunt, Uncle, Grandchild) ☐	b. No ☐

Family history of Diabetes

a. Yes ☐<br>i. 1st degree relations (Parents, Siblings, Child) ☐<br>ii. 2nd degree Relations (Grandparents, Aunt, Uncle, Grandchild) ☐	b. No ☐

Family history of Premature Cardiovascular disease or Stroke

a. Yes ☐<br>i. 1st degree relations (Parents, Siblings, Child) ☐<br>ii. 2nd degree Relations (Grandparents, Aunt, Uncle, Grandchild) ☐	b. No ☐

SECTION F: Anthropometric measurements, blood pressure, and blood parameters

Body weight (Kg)	
Height (m)	
Waist circumference (cm)	
Hip circumference (cm)	
Systolic blood pressure (mmHg)	
Diastolic blood pressure (mmHg)	
Fasting Plasma Glucose (mmol/L)	
Triglycerides (mmol/L)	
HDL-C (mmol/L)	
LDL-C (mmol/L)	
Total cholesterol (mmol/L)	
